# Determining the prevalence and predictors of sleep disordered breathing in patients with chronic heart failure: rationale and design of the SCHLA-HF registry

**DOI:** 10.1186/1471-2261-14-46

**Published:** 2014-04-09

**Authors:** Holger Woehrle, Olaf Oldenburg, Michael Arzt, Andrea Graml, Erland Erdmann, Helmut Teschler, Karl Wegscheider

**Affiliations:** 1 Sleep and Ventilation Center Blaubeuren, Respiratory Center Ulm, Ulm, Germany; 2 ResMed Science Center, Martinsried, Germany; 3 Department of Cardiology, Heart and Diabetes Center North Rhine-Westphalia, Bad Oeynhausen, Germany; 4 Department of Internal Medicine II, University Hospital Regensburg, Regensburg, Germany; 5 Clinic III for Internal Medicine, Heart Center University Hospital Cologne, Cologne, Germany; 6 Department of Pneumology, Respiratory and Sleep Medicine, Ruhrlandklinik, University Clinic Essen, Essen, Germany; 7 Department of Medical Biometry and Epidemiology, University Medical Center Hamburg-Eppendorf, Hamburg, Germany; 8 Schlafmedizinisches Zentrum, Klinik und Poliklinik für Innere Medizin II, Universitätsklinikum Regensburg, Franz-Josef-Strauss-Allee 11, 93053 Regensburg, Germany

**Keywords:** Registry, Prevalence, Heart failure, Sleep-disordered breathing, Obstructive sleep apnea, Central sleep apnea, Cheyne-Stokes respiration, Predictors

## Abstract

**Background:**

The objective of the SCHLA-HF registry is to investigate the prevalence of sleep-disordered breathing (SDB) in patients with chronic heart failure with reduced left ventricular systolic function (HF-REF) and to determine predictors of SDB in such patients.

**Methods:**

Cardiologists in private practices and in hospitals in Germany are asked to document patients with HF-REF into the prospective SCHLA-HF registry if they meet predefined inclusion and exclusion criteria. Screening was started in October 2007 and enrolment was completed at the end of May 2013. After enrolment in the registry, patients are screened for SDB. SDB screening is mainly undertaken using the validated 2-channel ApneaLink™ device (nasal flow and pulse oximetry; ResMed Ltd., Sydney, Australia). Patients with a significant number of apneas and hypopneas per hour recording time (AHI ≥15/h) and/or clinical symptoms suspicious of SDB will be referred to a cooperating sleep clinic for an attended in-lab polysomnography with certified scoring where the definite diagnosis and, if applicable, the differentiation between obstructive and central sleep apnea will be made. Suggested treatment will be documented.

**Discussion:**

Registries play an important role in facilitating advances in the understanding and management of cardiovascular disease. The SCHLA-HF registry will provide consistent data on a large group of patients with HF-REF that will help to answer questions on the prevalence, risk factors, gender differences and stability of SDB in these patients by cross-sectional analyses. Further insight into the development of SDB will be gained by extension of the registry to include longitudinal data.

## Background

The prevalence of heart failure (HF) in western countries is about 1-2% of the adult population, with significant increases as age increases [1,2]. Recent guidelines differentiate between HF due to reduced systolic left ventricular ejection fraction (HF-REF) and HF with preserved ejection fraction (HF-PEF) and impaired diastolic function [3]. HF-REF is the most widely investigated and best understood type of HF [3], with a high prevalence in males with ischemic heart disease. In contrast, HF-PEF is more prevalent in women and often has a non-ischemic aetiology. Epidemiological data suggest that HF-REF and HF-PEF have a similar prognostic impact [4].

A number of co-morbidities have been linked to the development and progression of HF. One that is gaining increasing recognition is sleep-disordered breathing (SDB) with predominant obstructive (OSA) or central sleep apnea (CSA) with or without Cheyne-Stokes respiration (CSR). Small studies published to date have reported that the prevalence of SDB was almost 70-80% in patients with HF-PEF and up to 76% in those with HF-REF based on a cut-off of an apnea-hypopnea index (apneas and hypoponeas per hour of recording time; AHI) ≥ 5/h while moderate to severe sleep apnea with an AHI ≥ 15/h was prevalent in about half of the patients [5-7].

The Sleep Heart Health Study identified OSA as an independent risk factor for the development of HF [8], with more impact in men than in women [9]. Patients with CSA have been shown to have a reduced quality of life [10] and to be at increased risk of developing cardiac arrhythmias [11]. In addition, the prevalence of CSA-CSR appears to increase as the severity of HF increases and cardiac function decreases [5,6]. Thus, the occurrence of CSA-CSR and its severity is thought to mirror cardiac dysfunction [12,13]. In addition, SDB in general [14], as well as OSA [15] and, in particular, CSA [16,17] have been shown to be independently associated with worse prognosis in patients with HF-REF. Even when HF-REF patients are receiving optimal treatment of HF, including cardiac resynchronization therapy (CRT), even a low level of CSA-CSR or OSA appears to have a major impact on prognosis [18].

Patient registries are increasingly being recognized as an important source of data about the natural history of disease, the effects of interventions or treatments, the relationship between baseline variables and outcomes, and the impact of risk factors [19-21]. Registries can also generate hypotheses to be tested in future randomized controlled clinical trials [19] and can serve as screening instruments for randomized trials. In this paper, the SCHLA-HF registry is introduced, which was started to fulfil these needs simultaneously for SDB in HF. For example, enrolment into the SCHLA-HF registry is a requirement for patients to be recruited into the SERVE-HF study (‘Treatment of Predominant Central Sleep Apnea by Adaptive Servo Ventilation in Patients with Heart Failure’; NCT00733343) [22]. SERVE-HF is the first multinational, multicentre, randomised, parallel trial to assess the effects of the addition of adaptive servo-ventilation (ASV) to optimal medical management compared with medical management alone on prognosis in HF-REF patients with predominant CSA. Its major substudy (NCT01164592) [22] will investigate potential mechanisms of benefit (e.g. reverse remodelling effects using echocardiography or changes in sleep using PSG). As screening instrument, the SCHLA-HF registry will include thousands of patients with stable HF-REF.

The objective of the registry is to investigate the prevalence of SDB in chronic HF-REF patients as well as risk factors, gender differences and stability of SDB in such patients. This report details data collection and assessments that will be undertaken in patients prospectively included in the SCHLA-HF registry.

## Methods

The SCHLA-HF registry received central ethics approval from the Freiburger Ethikkomission for Germany. All aspects of the registry were conducted within the principles of Good Clinical Practice and in accordance with the Declaration of Helsinki.

### Design of the registry

All German sleep laboratories were invited to participate in the registry. However, the majority did not have the infrastructure necessary to run such a project. Sleep laboratories were then asked to contact cardiologists in their referral area to build networks between cardiology and sleep medicine, which allowed the registry and the SERVE-HF study to proceed. Participating cardiologists, who had been contacted by and were working with sleep laboratories, were asked to enroll consecutively all heart failure patients who fulfilled the registry inclusion criteria described below.

**Figure 1:**
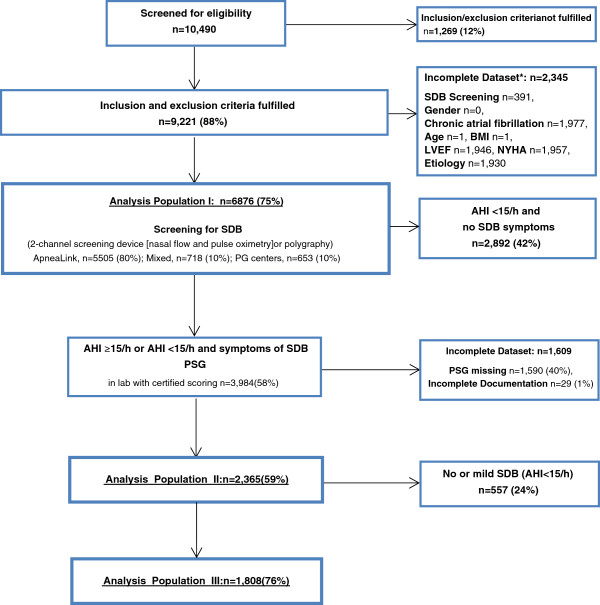
**Patient flow and populations in the SCHLA-HF registry.** AHI, apnea-hypopnea index; BMI, body mass index; LVEF, left ventricular ejection fraction; NYHA, New York Heart Association; PG, polygraphy; PSG, polysomnography; SDB, sleep-disordered breathing.

One hundred and thirty-eight centres in Germany (91 cardiology practices and 47 hospital cardiology departments) are testing patients with chronic HF-REF for eligibility (patient flow diagram, Figure 1). After enrolment in the registry, patients are screened for SDB. Patients with a significant number of apneas and hypopneas per hour recording time (AHI_screening_ ≥15/h) and/or clinical symptoms suspicious of SDB will be referred to one of the 66 cooperating sleep clinics for an attended in-lab polysomnography (PSG) with certified scoring where the definite diagnosis and will be made, and the suggested treatment will be documented (Figure 1). The registry was started in October 2007 and enrolment was completed at the end of May 2013. It is expected that data analysis will be complete by the end of 2014 and a final report available in 2015.

### Patients

For cardiologists screening patients, criteria for inclusion of patients in the SCHLA-HF registry are as follows: chronic HF diagnosed and treated according to the European Society of Cardiology (ESC) guidelines [23] ≥12 weeks prior to enrolment; moderate-to-severe left ventricular systolic dysfunction (left ventricular ejection fraction [LVEF] ≤45% by an imaging method such as echocardiography, radionuclide angiography, left ventriculography or cardiac magnetic resonance imaging) documented <12 weeks before enrolment; New York Heart Association (NYHA) class III or IV at the time of inclusion or NYHA class II with ≥1 hospitalisation for HF in the last 12 months; patient able to fully understand study information and give signed informed consent.

Patients are not eligible to be included in the registry if the had any of the following: current use of positive airway pressure (PAP) therapy; life expectancy <1 year for diseases unrelated to chronic HF-REF; cardiac surgery, percutaneous coronary intervention, myocardial infarction or unstable angina within 6 months prior to randomisation; CRT-implantation scheduled or performed within 6 months prior to randomisation; transient ischemic attack or stroke within 3 months prior to enrolment; primary hemodynamically-significant uncorrected valvular heart disease (obstructive or regurgitant) or any valvular disease expected to require surgery; acute myocarditis/pericarditis within 6 months prior to enrolment.

### Assessments

#### Demographic and clinical data

Demographic data, information about severity of HF-REF (New York Heart Association functional class, NYHA) and aetiology of HF-REF are assessed. In addition, common comorbidities of HF-REF (atrial fibrillation and diabetes) and symptoms of HF-REF/SDB such as nocturia, nap time, nocturnal dyspnea as well as medication are documented. Documentation of LVEF measured by an imaging method such as echocardiography, radionuclide angiography, left ventriculography or cardiac magnetic resonance imaging within 12 weeks prior to enrolment was added when data collection was expanded in May 2009, after 2395 patients had been enrolled. Also, the documentation of diabetes and heart rhythm was added.

#### Screening for sleep-disordered breathing

After enrolment in the registry, SDB screening is then undertaken mainly using the two channel screening system ApneaLink™ (ResMed Ltd., Sydney, Australia) which measures nasal flow via a cannula and records pulse oximetry that has been validated in several studies for screening of SDB [24]–[29]. The default settings of the screening device were used for the definitions of apnea, hypopnea and desaturation: apnea was defined as an 80% decrease in airflow for ≥10 seconds; hypopnea was defined as a decrease in airflow by 50-80% versus baseline for ≥10 seconds; desaturation was defined as a ≥4% decrease in oxygen saturation; SDB in the screening process was defined as AHI_screening_ ≥ 15/h.

In addition, the ApneaLink™ provides a flow-based classifier as an automated diagnostic test for CSR. The classifier of ApneaLink uses various features for identifying CSR. The main features are the cycle length (for CSR usually 45–90 s), the apnea-hypopnea length, the hyperpnea length, and the shape of the hyperpnea as described previously [30]. Of the 138 cardiology centres, 15 used respiratory polygraphy for screening of SDB, either alone (n = 7) or in combination with ApneaLink™. Apneas and hypopneas were defined and scored according to the criteria above.

Patients with SDB or typical clinical symptoms are being referred to a sleep laboratory where a polysomnography (PSG) will be performed to make a definite diagnosis of SDB.

#### Diagnosis of obstructive or central sleep apnea with or without Cheyne-Stokes Respiration

PSG will contain brain activity (electro encephalogram, EEG), eye movement (electro oculogram, EOG), muscle activity or skeletal muscle activation (electro myogram, EMG), heart rhythm (electro cardiogram, ECG), breathing and respiratory effort measures during sleep as well as the recording of body position. Flow measurement has to be performed by nasal cannula and thermistor during diagnostic procedures.

A standardized qualification process for each center has been used to ensure comparability between centers in the scoring of respiratory events. Prior to participation every center had to send two diagnostic respiratory scorings to a central lab that validated whether such scorings were performed according to a detailed operating manual for technical requirements of PSG and respiratory scoring. If quality was not acceptable, specific training was provided, followed by another quality assessment. A key element of the PSG scoring manual is the differentiation of apneas and hypopneas as central or obstructive using flattening of the inspiratory airflow curve, paradoxical breathing, arousal position, sleep stages, and breathing pattern at the end of the hypopnea [31,32].

### Statistical considerations

#### Sample size

The sample size of the SCHLA-HF registry is linked to the recruitment period of the SERVE-HF trial. No formal sample size calculations were performed for the registry. A total of three prespecified analysis datasets have been defined (Figure 1): the “Screening Data Set” (“Analysis Population I”), the “PSG Data Set” (Analysis Population II) and the “PSG AHI ≥15 Data Set” (Analysis Population III). Numbers presented are those up to end of May 2013 (Figure 1).

#### Statistical analysis

All variables will be analyzed using descriptive statistics (frequency, mean ± standard deviation [SD], range or median ± interquartile range, whichever is appropriate). Student’s t-test will be used to compare mean values. A Chi-square test will be used to compare frequencies and proportions in two or more groups. Data will be used for a nested case–control study comparing patients with and without SDB (analysis population I) or patients with different severity and type of SDB (analysis populations II and III) with respect to their characteristics and history. Multivariate logistic regression modelling will be used to analyze the associations between suspected causes of SDB and the risk of developing that disorder taking the cluster structure of the data entered by the recruiting cardiologists into account. The models will include potential clinical predictors of SDB, or of the extent of SDB, that are assessed in the SchlaHF registry such as age, BMI, gender, LVEF, NYHA class, aetiology of HF, and atrial fibrillation. Models will be fitted by likelihood ratio-based stepwise backward variable selection. The predictive accuracy of the resulting models will be judged by cross validation. A p value of <0.05 is considered to be statistically significant. Statistical analysis will be performed with SPSS version 19.0 or higher (SPSS Inc., Chicago, USA) or STATA 12.0 or higher (StataCorp LP, College Station, Texas, USA).

### Ethics and monitoring

The registry was approved by the local ethics committees of the participating institutions and is being managed in accordance with Good Clinical Practice and the Declaration of Helsinki. All patients provide written informed consent to be included in the registry. The patients are advised in the consent forms that they have the right to withdraw from the registry at any time without it impacting on their treatment. In the event that a patient drops out of the registry, a Registry Termination Form has to be completed, which details the date of termination and reason for termination. The patient is also informed she/he must specifically request that her/his data will not be analysed. Otherwise data remain in the database and will be included in the analysis. Reasonable effort will be made to contact any patient lost to follow-up (between SDB-screening and PSG) in order to complete assessments and retrieve any outstanding data.

## Discussion

Data from both large-scale clinical trials and registries have provided a large volume of data on patients with cardiovascular disease. It is increasingly recognised that large randomised, controlled clinical trials are extremely expensive to perform and often generate results that may not be generalizable to a wide population of patients in clinical practice [33]. Thus, the combination of good quality clinical studies and prospective registries is likely to be the best approach to identifying, defining and applying treatments and strategies to improve patient outcomes [19].

The focus of the SCHLA-HF registry is SDB in patients with HF-REF. SDB is increasingly being recognised as an independent risk factor for the development of HF [8,9,14,34,35] and as making an important contribution to worse outcomes in these patients [14-16]. In addition, worsening HF appears to increase the incidence and severity of SDB [5,6], while effective treatment of SDB appears to improve clinical outcomes in patients with HF [14,36].

One multicenter study and 5 single-center studies with ≥100 participants provide estimates of SDB prevalence in HF-REF (Table 1) [6,37-42]. Previous studies consistently indicate that the SDB is very common in HF. However, the SCHLA-HF registry may overcome some limitations of the previous studies. This will be achieved in a number of ways. Firstly, by including HF-REF patients from a large number of cardiology practices or hospital cardiology departments, the SCHLA-HF-registry will study a more representative sample compared to studies from specialized hospital cardiology departments [6,38,40,41] or sleep clinic populations [39] alone. Secondly, since the proportion of women with both HF-REF and SDB was low in previous studies, a very high total sample size, as targeted in the SCHLA-HF registry, is required to provide robust analyses of SDB prevalence and risk factors in women. Finally, in contrast to some of the previous studies [38-40], patients of the SCHLA-HF registry will be on current medical therapy for HF-REF.

**Table 1:** Prevalence of sleep-disordered breathing in chronic heart failure

**Study**	**Setting**	**Total patients (n)**	**Females (n)**	**SDB (AHI ≥15/h)**	**ß-blocker**	**Spironolactone**
Schulz et al. 2007 [37]	Multicenter	203	51	-*	90%	46%
Javaheri et al. 2006 [38]	Single center	100	0	49%	10%	0%
Oldenburg et al. 2007 [6]	Single center	700	139	51%	85%	62%
Sin et al. 1999 [39]	Single center	450	68	61%	0%	0%
Yumino et al. 2009 [40]	Single center	218	50	47%	75%	21%
Paulino et al. 2009 [41]	Single center	316	55	-*	82%	58%
Dolliner et al. 2013 [42]	Single center	176	26	50%	90%	60%

*reported prevalence of sleep-disordered breathing (SDB) based on a definition using apnea-hypopnea index cixAHI ≥10/h**.**

### Limitations

A multi-step procedure was established to improve the representativeness of the registry and, in particular, to capture as many SDB patients as possible, even if they were not accurately diagnosed at their first visit. However, such a multi-step procedure can itself be a source of bias. Perhaps the most important potential limitation of this study is the interaction/collaboration between cardiology physicians and the sleep laboratories. Such interactions have the potential to introduce bias, and missing PSG results need to be taken into account when interpreting the data.

### Perspective

Effective management of SDB and associated improvements in HF morbidity and mortality is a potential approach to managing the many HF patients who remain symptomatic and have disease progression despite guideline-driven pharmacological and device-based therapy. It is hoped that data gathered by the SCHLA-HF registry will make a substantial contribution to the body of knowledge in the area of HF-REF and SDB. In addition, it has the potential to generate rational hypotheses with respect to the sequence of symptoms, and the aetiology and potential treatment of the disease to be tested in future randomised clinical trials, which have been called for to develop quality treatment guidelines [43]. Hopefully this will lead to better strategies to manage HF, particularly with respect to effective treatment of SDB, which in turn will contribute to improved morbidity and mortality outcomes in these patients.

## Conclusions

Registries play an important role in facilitating advances in the understanding and management of cardiovascular disease. The SCHLA-HF registry will provide consistent data on a large group of patients with HF-REF that will demonstrate aspects of the natural course of the disease and help to answer questions on the prevalence, risk factors, gender differences and stability of SDB in these patients.

## Abbreviations

AHI: Apnea-hypopnea index; ASV: Adaptive servo-ventilation; BMI: Body mass index; CRT: Cardiac resychronization therapy; CSA: Central sleep apnea; CSR: Cheyne-Stokes respiration; HF: Heart failure; HF-REF: Heart failure with reduced systolic function; HF-PEF: Heart failure with preserved ejection fraction; LVEF: Left ventricular ejection fraction; NYHA: New York Heart Association; OSA: Obstructive sleep apnea; PAP: Positive airway pressure; PSG: Polysomnography; SDB: Sleep-disordered breathing.

## Competing interests

The SCHLA-HF registry was funded by ResMed Ltd, Sydney, Australia and ResMed Germany Inc, Martinsried, Germany. MA received grant support from ResMed (Martinsried, Germany), Philips Home Healthcare Solutions (Murrysville, PA, USA), and the German Foundation for Cardiac Research (Deutsche Stiftung für Herzforschung); MA is the holder of an endowed professorship from the Free State of Bavaria at the University of Regensburg that was donated by ResMed (Martinsried, Germany) and Philips Home Healthcare Solutions (Murrysville, PA, USA); MA has previously received lecture fees from AstraZeneca, Philips Home Healthcare Solutions (Murrysville, PA, USA) and ResMed (Martinsried, Germany). HT received grant support from ResMed (Sydney, Australia), the ResMed Foundation (San Diego, USA) and Linde, Munich, Germany. HT has previously received lecture fees from AstraZeneca, Novartis, Linde, Boehringer Ingelheim, Berlin Chemie, and ResMed Germany. OO has acted as a consult for ResMed and Respicardia, received a research grant from ResMed, and has received lecture honoraria from ResMed and Weinmann. EE and KW received consulting fees or honoraria, and travel grants, from ResMed. HW and AG are employees of ResMed, Germany.

## Authors’ contributions

HW was involved in the conception, hypotheses delineation, and design of the study, the analysis and interpretation of such information, writing the article and in its revision prior to submission. OO, MA, EE, and HT were substantially involved in the design of the study, acquisition and interpretation of the data, and critical revision of the article prior to submission. AG performed parts of the statistical analysis and helped to draft the manuscript. KW participated in the design of the study, in particular the statistics, performed parts of the statistical analysis and helped to draft the manuscript. All authors read and approved the final manuscript.

## Pre-publication history

The pre-publication history for this paper can be accessed here:

http://www.biomedcentral.com/1471-2261/14/46/prepub
